# Effects of a hydroalcoholic extract of *Juglans regia* (walnut) leaves on blood glucose and major cardiovascular risk factors in type 2 diabetic patients: a double-blind, placebo-controlled clinical trial

**DOI:** 10.1186/s12906-018-2268-8

**Published:** 2018-07-04

**Authors:** Khadijeh Rabiei, Mohammad Ali Ebrahimzadeh, Majid Saeedi, Adele Bahar, Ozra Akha, Zahra Kashi

**Affiliations:** 1 World Federation of Acupunture-Moxibustion Societies (WFAS), Scientific Studies Institute of Nadali Esmaeili, Acupuncture Center, Sari, Iran; 2 Pharmaceutical Sciences Research Center, Hemoglobinopathy Institute, Mazandaran University of Medical Sciences, Sari, Iran; 3 Diabetes Research Center, Mazandaran University of Medical Sciences, Sari, Iran; 4 Traditional and Complementary Medicine Research Center, Addiction Institute, Mazandaran University of Medical Sciences, Sari, Iran

**Keywords:** Diabetes mellitus, *Juglans regia*, Walnut leaves, Herbal medicine, Weight, Blood glucose, Blood pressure, Cardiovascular, Microcrystalline cellulose, Avicel

## Abstract

**Background:**

We aimed to evaluate the effects of a hydroalcoholic extract of *Juglans regia L.* leaves on blood glucose level and cardiovascular risk factors in type 2 diabetic patients.

**Methods:**

In this randomized, double-blind, placebo-controlled, parallel-group (2 arms) clinical trial, 50 diabetic patients were divided into two groups: treatment group (receive the capsules containing 100 mg *J. regia* leaf extract) and control group (receive the capsules containing placebo, microcrystallin cellulose). Baseline participant data were matched between the two arms of the study. We administered the prepared capsules to the patients twice daily for 8 weeks. Blood glucose level, glycosylated hemoglobin (HbA1c) level, body weight, body mass index, blood pressure, lipid profile, serum insulin, and insulin resistance were compared between the two groups before and after the intervention. *P* < 0.05 was considered significant.

**Results:**

After excluding eleven patients, 20 received *J. regia* leaf extract and 20 patients received placebo. The *J. regia* leaf extract did not significantly change the blood glucose and insulin resistance condition. However, in this group, body weight, body mass index, and systolic blood pressure significantly decreased compared with the baseline measurements (*P* = 0.028, *P* = 0.030, and *P* = 0.005, respectively). The lipid profile did not change significantly compared with the baseline measurements. In the control group, postprandial glucose and HbA1c levels significantly decreased after the intervention (P = 0.030 and P = 0.028, respectively). The other variables were not significantly different in this group. At the end of the study, the variables were not significantly different between the two groups.

**Conclusion:**

In this double-blind study, 200 mg/d of *J. regia* leaf extract had no significant effect on blood glucose level and HOMA-IR score in patients with type 2 diabetes. However, the *J. regia* leaf extract was effective in reducing body weight and blood pressure. An accidental finding of our study was that microcrystalline cellulose, a widely used placebo in clinical trials, led to a reduction in blood glucose level.

**Trial registration:**

Iranian Registry of Clinical Trials (IRCT: 138901203180 N2, 2010/6/6); retrospectively registered.

## Background

Diabetes mellitus is an important metabolic disease and the most prevalent chronic illness around the world with a high financial burden. According to the World Health Organization report, 422 million people have diabetes worldwide, and this rate is rising rapidly [1].

Diet, exercise, and medications are used to manage the disease. However, most patients are reluctant to use chemical drugs, and sometimes, they do not achieve adequate disease control despite the use of multiple medications [2]. The management of cardiovascular risk factors, including weight, lipid levels, and blood pressure, in addition to blood glucose, is very important in patients with type 2 diabetes mellitus [3]. The available anti-diabetic drugs have different effects on the cardiovascular risk factors in diabetic patients, with some of them having a positive effect and some a negative effect. Currently, researchers are giving special attention to the use of medicinal herbs for the treatment of various diseases. The leaves of *Juglans regia L.* (*J. regia*) have been used in traditional medicines as an antimicrobial, anthelmintic, keratolytic, and antidiarrheal and are rich in polyphenolic compounds and flavonoids [4] Amongst the several categories of phytochemicals, polyphenols are the most attractive ones, especially for medicinal purposes [5]. Polyphenols are an important class of secondary metabolites of the plant, possessing a variety of pharmacological activities. Plant phenolics are multifunctional and can act as reducing agents, metal chelators, and singlet oxygen quenchers [6]. Several polyphenols have been shown to have significant antioxidant activities through in vitro and in vivo studies [5]. Studies have shown that consumption of foods and beverages rich in phenolic content is correlated with a reduced risk of atherosclerosis and cardiovascular disease [7]. Literature review shows that polyphenols have demonstrated beneficial effects in animal models of several cardiovascular disorders like hypertension, atherosclerosis, endothelial dysfunction, dyslipidemia, and diabetes-related cardiovascular complications [5] Flavonoids form a ubiquitous group of polyphenolic substances typically produced by plants. Flavonoids are of great interest because of their bioactivities, which are basically related to their antioxidant properties [8]. It has been recognized that flavonoids show antioxidant activity, and their effects on human nutrition and health are considerable. Flavonoids may slow the pathogenesis of atherosclerosis and cardiovascular diseases by their ROS scavenging effects. The mechanism of action of flavonoids involves a scavenging or chelating process [8].

Some studies have reported the anti-diabetic effects of *J. regia* leaves in rats [9–11]; nevertheless, the number of human studies is few. The present study was designed to evaluate the effect of a hydroalcoholic extract of *J. regia* leaves on hyperglycemia and cardiovascular risk factors in patients with type 2 diabetes mellitus.

## Methods

This study was a randomized, double-blind, placebo-controlled, parallel group clinical trial. We evaluated the effect of a hydroalcoholic extract of *J. regia* leaves on blood glucose as the primary outcome and insulin resistance, lipid profile, blood pressure, and body weight, the cardiac risk factors, as the secondary outcomes in type 2 diabetic patients.

### Preparation of the formulations plant material

*J. regia* (Juglandaceae) leaf was collected from Dashtenaz area, Sari, Iran. After identification by Dr. Bahman Eslami (Assistance professor of plant systematic, Islamic Azad University, Branch of Ghaemshahr, Iran), Voucher specimen (No 629) was deposited in the Sari School of Pharmacy. The sample was dried at room temperature on the ground before extraction. One kilogram of the sample was extracted by percolation with 70% ethanol (2.5 L × 3) for 24 h [12–14]. The resultant extracts were concentrated in a rotary evaporator until a solid crude extract was obtained, which was freeze-dried to remove the solvent (15.5%) completely. The dried extracts were powdered and mixed with microcrystalline cellulose (Avicel) and then were encapsulated. Avicel was purchased from Sigma-Aldrich (USA). Each capsule contained 100 mg extract and 400 mg Avicel. Avicel itself was used as placebo. Each placebo capsule contained 500 mg Avicel. The final formulations were controlled microbiologically based on the United States Pharmacopeia (USP) method [15].

### Standardization of extract

The extract was standardized based on the phenol content. The total phenolic content was determined using the Folin–Ciocalteu method [16] . Each capsule contained 40 mg ± 1.3 mg gallic acid equivalent per gram extract.

### Study design

The participants in the study were selected from among those referred to the diabetes outpatient academic clinic in Imam Khomeini Hospital, Sari, Iran (2012–2013). The inclusion criteria were an age of 30–80 years and glycosylated hemoglobin (HbA1c) level more than 7% in spite of receiving the maximal dose of two anti-diabetic drugs (metformin and glibenclamide). Patients were not included in the study if they had immunodeficiency, uncontrolled thyroid dysfunction, cardiovascular disease, proliferative retinopathy, acute hepatitis or cirrhosis, acute infection, history of diabetic ketoacidosis, severe weight loss (at least 10% during the past 6 months), current corticosteroid or thiazide consumption, and serum creatinine (Cr) level > 1.5 mg/dl in males and > 1.4 mg/dl in females. The exclusion criteria also included pregnancy, lactation, changes in anti-diabetic drug type, and lack of follow-up.

Fifty eligible type 2 diabetic patients were enrolled in the study. The minimum sample size was determined to be 20 patients in each group for a statistical power of 0.8 and 95% confidence level and treatment effect size of 0.5–0.8 decrease in the HbA1C following the intervention. After being explained about the trial, the patients signed an informed consent form and were randomly (manual methods and sequentially numbered envelopes) divided into two groups (by a trained general physician who was blind to the content of the capsules), *J. regia* leaves group or the placebo capsule group. The fasting blood sugar (FBS), postprandial glucose (PPG), HbA1c, HOMA-IR, body weight, and blood pressure (BP) were not different between the two groups at baseline. (Table 1) All the patients were advised not to change their previous medications and standard diet during the study period.

**Table 1:** Baseline participant data in two arms, *Juglans regia* leaves group and control group (*n* = 50)

Variable	*Juglans regia* leaves group n (*N* = 25) Mean ± SD	placebo group (*N* = 25) Mean ± SD	*P* value
Weight (kg)	74.5 ± 15.4	73.1 ± 9.2	0.744
BMI (kg/cm^2^)	29.7 ± 5.8	30.2 ± 3.8	0.738
Systolic blood pressure (mm Hg)	126.4 ± 9.1	121.7 ± 10.5	0.153
Diastolic blood pressure (mm Hg)	79.7 ± 6.7	76.7 ± 9.8	0.275
Fasting blood glucose (mg/dl)	195.2 ± 38.2	205 ± 51.9	0.500
Postprandial blood glucose (mg/dl)	283.8 ± 45.5	303.2 ± 68.1	0.323
HbA1C (%)	9.6 ± 1.1	9.8 ± 0.8	0.421
Insulin level	7.2 ± 5	6.4 ± 3.2	0.598
HOMA IR	3.6 ± 2.9	2.9 ± 1.6	0.451
Creatinine (mg/dl)	0.8 ± 0.1	0.9 ± 0.2	0.671
Hemoglobin (mg/dl)	12.8 ± 1.8	12.4 ± 1.3	0.458
Cholesterol (mg/dl)	176.4 ± 39.7	183.4 ± 31.5	0.547
Triglyceride (mg/dl)	180 ± 81.7	167.8 ± 76.6	0.631
HDL Cholesterol (mg/dl)	49.3 ± 8.9	46 ± 12.8	0.336
LDL Cholesterol (mg/dl)	92.5 ± 30.1	102.2 ± 22.3	0.257
AST (U/L)	18.9 ± 4	24.4 ± 9.7	0.022
ALT (U/L)	20 ± 6.2	25.2 ± 13	0.114
ALP (IU/L)	196.9 ± 59.1	214.1 ± 55.1	0.347
TSH (IU/L)	2.2 ± 1.2	2.4 ± 0.9	0.716

The drug or placebo was administered by a trained physician once per day for 1 week and then twice per day for 7 weeks. Body weight, body mass index (BMI), BP, FBS, serum insulin, HbA1c, PPG, serum lipid profile, and liver function were measured at baseline and after treatment. A general physician, who was blinded to the treatment type, examined the patients after 2 weeks and 8 weeks and counted the number of the remaining capsules for the assessment of the participants’ adherence to the interventions. Insulin resistance was calculated using the homeostasis model assessment-estimated insulin resistance (HOMA-IR) method [17] .

$$ {HOMA}_{IR}=\frac{fasting\ plasma\ insulin\kern0.75em \raisebox{1ex}{$\mathrm{mIU}$}\!\left/ \!\raisebox{-1ex}{$\mathrm{L}$}\right.\times fasting\ plasma\ glucose\kern0.5em \ \raisebox{1ex}{$\mathrm{mmol}$}\!\left/ \!\raisebox{-1ex}{$\mathrm{L}$}\right.}{22.5} $$

### Statistical analysis

The analysis was according to the original assigned groups. Student’s t-test and paired t-test were used to compare the quantitative variables between the two groups and the before-after values of each group, respectively. The qualitative variables were compared between the two groups using Chi-square test and Fisher exact test, if necessary. In all calculations, *P* < 0.05 was considered to be significant.

## Results

**Fig. 1:**
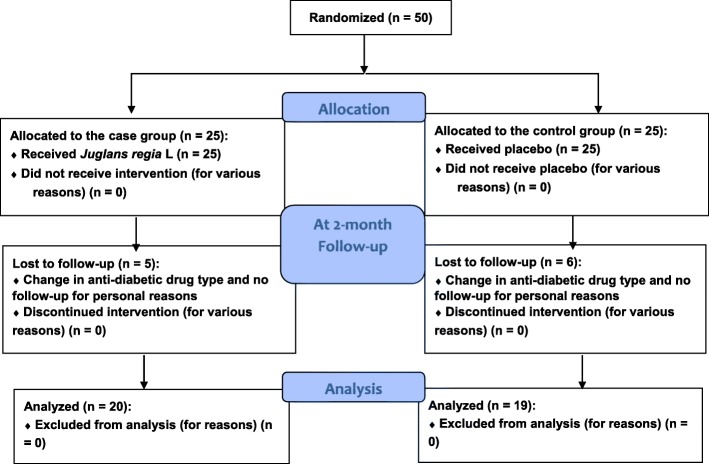
Study flow diagram based on the CONSORT 2010 flow diagram

In this study, 25 patients were assigned to the intervention group, and 25 patients were assigned to the control group (2012 until 2013). However, because of the changes in the anti-diabetic drug type or lack of follow-up, five patients in the extract group and six patients in the control group were excluded from the study. Fig 1.

The mean age of the patients was not significantly different between the intervention group (50.5 ± 8.3 years) and the control group (49.9 ± 8.6 years) (*P* = 0.84). There was a history of hypertension and dyslipidemia in 52.4 and 85.7% of the participants in the intervention group, respectively, and 42.1 and 84.2% in the control group, respectively (*P* = 0.5 for hypertension and *P* = 0.9 for dyslipidemia). Most of the participants were female; there were 18 females in the control group and 19 females in the intervention group (*P* = 0.08).

The *J. regia* leaves extract had no significant effect on the FBS, PGG, HbA1c level, insulin resistance, and lipid profile, but *J. regia* leaves extract significantly decreased the body weight, BMI, and systolic BP compared with the baseline measurements (*P* = 0.028, *P* = 0.030, and *P* = 0.005, respectively). In the control group, the PGG, HbA1c, and alanine transaminase (ALT) levels decreased significantly after the intervention (P = 0.030, P = 0.028 and *P* = 0.044, respectively). Although the aspartate transaminase (AST) level was higher in the placebo group at baseline, there were no significant differences in any of the variables between the two groups at the end of the study (Table 2). The participants in the treatment and control group reported no side effects.

**Table 2:** Participant data before and after intervention in the case group (extract of *Juglans regia* leaves) and the placebo group (Avicel)

Variable	Juglans regia leaves group┼ (*N* = 20)		*P* value	placebo group (*N* = 19)		*P* value
	Before intervention Mean ± SD	After intervention Mean ± SD		Before intervention Mean ± SD	After intervention Mean ± SD	
Weight (kg)	73.0 ± 15.1	**71.7 ± 13.8**	**0.028**	73.2 ± 9.2	72.3 ± 8.7	0.303
BMI (kg/cm^2^)	29.2 ± 6.0	**28.7 ± 5.3**	**0.030**	30.3 ± 3.9	29.9 ± 4.0	0.336
Systolic blood pressure (mm Hg)	126.1 ± 9.5	**121.1 ± 8.8**	**0.005**	121.8 ± 10.6	120.6 ± 9.7	0.637
Diastolic blood pressure (mm Hg)	79.2 ± 6.7	77.4 ± 4.8	0.185	76.8 ± 9.8	79.7 ± 4.8	0.116
Fasting blood glucose (mg/dl)	191.7 ± 36.6	179.5 ± 49.0	0.309	205.1 ± 51.9	194.6 ± 64.8	0.447
Postprandial blood glucose (mg/dl)	283.2 ± 46.8	307.7 ± 99.0	0.249	303.2 ± 68.2	**255.4 ± 53.7**	**0.030**
HbA1C (%)	9.6 ± 1.1	9.5 ± 1.8	0.646	9.90 ± 0.9	**9.1 ± 1.3**	**0.028**
Insulin level	6.4 ± 3.7	7.3 ± 3.9	0.447	6.2 ± 3.3	4.5 ± 3.1	0.139
HOMA IR	3.3 ± 2.7	2.9 ± 2.2	0.186	3.0 ± 1.7	2.7 ± 1.4	0.395
Creatinine (mg/dl)	0.9 ± 0.2	0.8 ± 0.2	0.474	0.9 ± 0.2	0.8 ± 0.2	0.270
Hemoglobin (mg/dl)	12.8 ± 1.9	13.2 ± 2.0	0.119	12.5 ± 1.3	12.3 ± 1.2	0.601
Cholesterol (mg/dl)	176.5 ± 41.8	169.0 ± 30.5	0.413	183.4 ± 31.6	176.5 ± 35.8	0.495
Triglyceride (mg/dl)	179.7 ± 86.1	170.6 ± 81.9	0.622	167.8 ± 76.7	184.1 ± 105.6	0.276
HDL Cholesterol (mg/dl)	49.0 ± 9.3	51.1 ± 9.1	0.337	44.9 ± 12.2	46.2 ± 13.6	0.710
LDL Cholesterol (mg/dl)	93.2 ± 31.6	83.5 ± 16.0	0.151	102.3 ± 22.3	93.0 ± 15.7	0.187
AST (U/L)	19.1 ± 4.2	19.2 ± 6.2	0.902	23.9 ± 9.6	19.4 ± 6.7	0.056
ALT (U/L)	20.7 ± 6.5	20.2 ± 8.1	0.803	24.7 ± 12.4	**18.3 ± 5.7**	**0.044**
ALP (IU/L)	200.7 ± 61.3	197.4 ± 68.0	0.817	202.9 ± 51.1	194..6 ± 64.2	0.594
TSH (IU/L)	2.2 ± 1.2	–	–	2.4 ± 0.9	–	–

┼ There were no significant differences in any of the variables between the two groups at the end of the study

These entries are in boldface because these variables significantly changed after internention (*p* value <0.05)

## Discussion

The use of *J. regia* leaves for the management of diabetes mellitus has been described in the Iranian traditional medicine [18].

In the present study, the *J. regia* leaves had no significant effect on the blood glucose and HOMA-IR levels in the diabetic patients. However, the leaf extract significantly decreased the body weight and systolic BP without any adverse effects on the liver and kidney function.

According to the literature on traditional medicines, the herbal medicines and their extracts are useful in the treatment of chronic disorders, including diabetes mellitus. The herbal medicines have a protective and therapeutic effect in diabetes mellitus via regeneration of the pancreatic β cell, glycogen degradation, decreased gluconeogenesis, α-glucosidase enzyme inhibitor activity, and antioxidative stress [19, 20].

Some previous studies investigated the effect of *J. regia* leaves in rats and showed that it had a positive effect on the blood glucose level. They reported that the hypercellularity of the pancreatic islet tissue was associated with increased hyperchromic nucleus of the islet cells. This finding may be indicative of regeneration of the beta cells [9]. According to the study by Kamyab et al. [21] in mice, oral walnut leaf and ridge extracts significantly reduced liver pyruvate carboxykinase activity and increased liver glycogen phosphorylase activity. They concluded that walnut could reduce the blood glucose level by inhibiting hepatic gluconeogenesis and stimulating secretion of pancreatic insulin.

There are a few scientific studies on the anti-diabetic effect of *J. regia* leaves in humans. We found only two human studies that looked at the effect of *J. regia* leaves in patients with type 2 diabetes [13, 22] . Both studies were done in Iran and reported the significant effect of this plant on blood glucose and insulin levels. However, in the study by Hosseini et al. [13], the plant extract was not standardized, and it was not clear what the placebo was. Also, the researchers prepared the plant extract and placebo in a tablet form, which can affect the double blinding of the study because of the smell of walnut. In a study by Abdoli et al. [22], the aqueous extract of *J. regia* leaves had significant blood glucose lowering effect in patients with type 2 diabetes. The baseline fasting plasma glucose was significantly lower than that in the control group in the study by Abdoli et al. Also, toast powder was used as the placebo in this study, which may itself increase the blood glucose levels [23].

In the present study, we used microcrystalline cellulose (Avicel) as the placebo. Microcrystalline cellulose is an insoluble fiber. When given orally, the agent is not absorbed and has no toxicity; therefore, it is widely used as a placebo in clinical trials [24, 25]. Surprisingly, in our study, low dose of Avicel taken orally (1000 mg/day) significantly lowered the PPG and HbA1c. We did not find any published article on the effect of microcrystalline cellulose (Avicel) on blood glucose in humans. In the study by Takahashi et al. [23] in rats, the consumption of cellulose with meals increased the digestive viscosity and modulated the postprandial plasma glucose.

In our study, the extract of *J. regia* leaves had a significant effect on the body weight and BP. Neither Hosseini et al. [13] nor Abdoli et al. [22] reported about the effect of the extract of *J. regia* leaves on the body weight or BP. Ma et al. [26]. evaluated the effects of a walnut-enriched diet on the endothelial function in patients with type 2 diabetes and reported a significant improvement in the endothelial function and BP. However, the walnut-enriched diet had no significant effect on the blood glucose, HbA1c, and insulin sensitivity. In our study, the consumption of *J. regia* leaves extract led to a significant reduction in the body weight. This effect was also reported by Rock et al. [27] in obese men and women who were given a walnut-enriched diet. We did not find a positive or negative effect of *J. regia* leaves on the lipid profile. Though Hosseini et al. [13] reported the hypolipidemic effects of *J. regia* leaves, Abdoli et al. [22] did not find a positive effect similar to our study. In the study by Ma et al., the walnut-enriched diet had no significant effect on lipid profile.

One of the limitations of our study was that most of the patients were female, although gender did not appear to have a significant effect on the participants’ response to the plant extract [28]. Another limitation was the short duration of our study.

## Conclusion

The main finding of our study is that the *J. regia* leaves extract is effective in decreasing some major cardiovascular risk factors including body weight and BP in patients with type 2 diabetes. However, the extract had no significant effect on the blood glucose, HOMA-IR, and lipid profile. An accidental finding of our study was that microcrystalline cellulose, which is widely used as a placebo in clinical trials, led to a reduction in the blood glucose level, particularly the PPG. The *J. regia* leaves had no side effects and were safe in low dose (200 mg/d) in our study. These results can be important for researchers who want use this agent as a placebo in clinical trials. On the other hand, this finding may be the first step for future studies to use this substance as a hypoglycemic drug.

## Abbreviations

ALT: Alanine transaminase
AST: Aspartate aminotransferase
BMI: body mass index
BP: blood pressure
Cr: Creatinine
FBS: Fasting blood sugar
HbA1c: glycosylated hemoglobin
HOMA-IR: homeostasis model assessment method–insulin resistance
*J. regia*: *Juglans regia L*.
PPG: postprandial blood glucose
USP: United States pharmacopeia
